# Densities of *Agrilus auroguttatus* and Other Borers in California and Arizona Oaks

**DOI:** 10.3390/insects5010287

**Published:** 2014-03-21

**Authors:** Laurel J. Haavik, Tom W. Coleman, Mary Louise Flint, Robert C. Venette, Steven J. Seybold

**Affiliations:** 1 Department of Entomology, University of California-Davis, One Shields Avenue, Davis, CA 95616, USA; E-Mail: mlflint@ucdavis.edu; 2 USDA Forest Service, Forest Health Protection, 602 S. Tippecanoe Avenue, San Bernardino, CA 92408, USA; E-Mail: twcoleman@fs.fed.us; 3 USDA Forest Service, Northern Research Station, 1561 Lindig Street, St. Paul, MN 55108, USA; E-Mail: rvenette@fs.fed.us; 4 USDA Forest Service, Pacific Southwest Research Station, HDH001 Orchard Park Drive, Rm 116, Davis, CA 95616, USA; E-Mail: sjseybold59@gmail.com

**Keywords:** host suitability, invasive species, oak ecosystems, phloem and wood borer, population dynamics

## Abstract

We investigated within-tree population density of a new invasive species in southern California, the goldspotted oak borer, *Agrilus auroguttatus* Schaeffer (Coleoptera: Buprestidae), with respect to host species and the community of other borers present. We measured emergence hole densities of *A. auroguttatus* and other borers on the lower stem (bole) of naïve oaks at 18 sites in southern California and on co-evolved oaks at seven sites in southeastern Arizona. We sampled recently dead oaks in an effort to quantify the community of primary and secondary borers associated with mortality—species that were likely to interact with *A. auroguttatus*. Red oaks (Section *Lobatae*) produced greater densities of *A. auroguttatus* than white oaks (Section *Quercus*). On red oaks, *A. auroguttatus* significantly outnumbered native borers in California (mean ± SE of 9.6 ± 0.7 *versus* 4.5 ± 0.6 emergence holes per 0.09 m^2^ of bark surface), yet this was not the case in Arizona (0.9 ± 0.2 *versus* 1.1 ± 0.2 emergence holes per 0.09 m^2^). In California, a species that is taxonomically intermediate between red and white oaks, *Quercus chrysolepis* (Section *Protobalanus*), exhibited similar *A. auroguttatus* emergence densities compared with a co-occurring red oak, *Q. kelloggii*. As an invasive species in California, *A. auroguttatus* may affect the community of native borers (mainly Buprestidae and Cerambycidae) that feed on the lower boles of oaks, although it remains unclear whether its impact will be positive or negative.

## 1. Introduction

The goldspotted oak borer, *Agrilus auroguttatus* Schaeffer (Coleoptera: Buprestidae), is a new pest of oaks in southern California (CA) [1]. It was first collected in CA in 2004 [2]. Collection records throughout the 20th century indicate that *A. auroguttatus* is native to mountain ranges in southeastern Arizona (AZ) [3]. Current genetic evidence is consistent with AZ populations as the probable source for CA populations [4]. Prior to its discovery in CA, little was known about this beetle’s biology and ecology. Knowledge of the effect of host species [5,6] and interactions with the community of other borers present within its native and introduced ranges are important for understanding the invasion biology of *A. auroguttatus*.

In their new ecosystems, invasive species populations can reach damaging levels because new habitats may lack important natural enemies (e.g., enemy release, [7]), or co-evolved resistance mechanisms among new host plants (e.g., biotic resistance, [8]). Consequently, invasive insects can have direct effects on populations of these naïve host plants and indirect effects on native populations of specialist herbivores that may share hosts with the invader [9,10,11]. Comparing the biology and ecology of invasive species in their new habitats with those in their native ranges can further understanding of invasion biology and may help to identify possible management techniques for invasive pests. 

Direct comparisons of *A. auroguttatus* population densities on species from the red (Section *Lobatae*) and white oak (Section *Quercus*) groups in CA or AZ have not been made among individual trees of similar physiological status. Such comparisons may confirm whether red oaks are more preferable or suitable hosts than white oaks for *A. auroguttatus*. Thus far, extensive mortality (approx. 25,000 dead trees) in southern CA has occurred primarily among two species in the red oak group: CA black oak, *Quercus kelloggii* Newb., and coast live oak, *Q. agrifolia* Née [12]. Known host species of *A. auroguttatus* in AZ (also in Section *Lobatae*) include Emory oak, *Q. emoryi* Torrey, and silverleaf oak, *Q. hypoleucoides* A. Camus [3]. In CA, *A. auroguttatus* also colonizes the taxonomically intermediate species canyon live oak, *Q. chrysolepis* Liebm. (Section *Protobalanus*) and Engelmann oak, *Q. engelmannii* Greene, from the white oak group, although the latter is colonized at very low densities [13,14]. There have been no observations of *A. auroguttatus* on Arizona white oak, *Q. arizonica* L. (Section *Quercus*), which co-occurs with its red oak hosts in AZ [3,13]. 

*Agrilus auroguttatus* is the primary biotic agent causing tree mortality in southern CA oak forests [13], but its influence on native borer populations in the main stem of oaks remains largely unknown. Comparisons of *A. auroguttatus* population densities to densities of other borers may be a first step towards determining borer community interactions in oak ecosystems. A suite of native oak insects feed in subcortical tissues within the main stem (bole) of southern CA oaks [15,16,17]. Records of oak-feeding insects in southeastern AZ are more limited [16,18]. 

Our objective was to investigate within-tree densities of *A. auroguttatus* in its introduced and native ranges with respect to host species and the community of other borers present. We compared the density of emergence holes from *A. auroguttatus* and other borers on different species of recently dead oaks among several sites in CA and AZ. Our study focused on the community of borers in the lower bole because *A. auroguttatus* occurs most frequently in this portion of the stem [19]. We hypothesized that: (1) species in the red oak group would be better hosts for *A. auroguttatus*, and thus produce higher emergence densities than co-occurring white oak species; and (2) *A. auroguttatus* would outnumber other borers in its introduced range (CA), but not in its native range (AZ). 

## 2. Materials and Methods

### 2.1. Study Sites: Southern California

Eighteen study sites were selected in the current zone of *A. auroguttatus* infestation within the Cleveland National Forest and one site was selected outside the currently infested zone (Warner Springs) in southern CA (Table 1; infestation zone as of 2010 in [3]). Sites were distributed approximately evenly throughout the public forested lands of the Descanso Ranger District to evaluate variation in borer populations throughout the National Forest. Ten sites were *Q. agrifolia*-dominant oak woodlands (900–1500 m in elevation) and the remaining nine sites were mixed *Q. kelloggii*-pine forests (mostly *Pinus jeffreyi* Grev. & Balf.; 1500–1800 m in elevation). We searched for, but could not find, sites where both of these red oak species occurred and *A. auroguttatus* was present. Thus, statistical comparisons of borer density between these two host species were not possible because the effect of host species on *A. auroguttatus* density was confounded with the effects of location and elevation. Comparisons of *A. auroguttatus* emergence holes could only be made between host species pairs at two sites (Roberts’ Ranch and Thing Valley), where two red oak/non-red oak species pairs occurred (Table 1).

Climate in the Descanso Ranger District is Mediterranean with hot summers (mean August and January maximum temperatures: 30 °C and 14 °C, respectively [20]) [21]. The majority of precipitation occurs in the winter (total annual precipitation is 53 cm [20]). Soils are rocky, well-drained sandy loams above decomposed granitic bedrock [21].

### 2.2. Study Sites: Southeastern Arizona

Seven study sites were selected within the Coronado National Forest in southeastern AZ where oak mortality was identified during previous surveys [13]. Sites with *Q. emoryi* were oak woodlands (1400–1700 m elevation) and sites with *Q. hypoleuocides* and *Q. arizonica* were mixed oak-pine forests that also contained *Juniperus deppeana*, *Pinus* spp., *Platanus racemosa* Nutt., and *Acer* spp. (1500–1750 m elevation) (Table 1). It was difficult to find sites in southeastern AZ with both co-occurring oak species and with evidence of *A. auroguttatus* infestation. Only one study site, Cochise Stronghold Campground, contained all three oak species. Borer densities were also compared between *Q. arizonica* and *Q. hypoleucoides* at Carr and Miller Canyons. At all other sites, borer emergence holes were counted on only one oak species.

**Table 1:** Site information, tree sample sizes (n) and tree size (diameter at breast height, DBH) by host species and taxonomic section.

Location	Site names	Host species (Section)	Coordinates (°N, °W)	Elevation (m)	n	DBH ^a^ (cm)
CA	Roberts’ Ranch	*Q. agrifolia* (*Lobatae*)/*Q. engelmannii* (*Quercus*)	32.82920, 116.61699	1051	17	74.2 ± 8.2
	Thing Valley	*Q. kelloggii* (*Lobatae*)/*Q. chrysolepis* (*Protobalanus*)	32.85440, 116.42055	1801	20	72.0 ± 6.9
	Cuyamaca State Park	*Q. agrifolia* (*Lobatae*)	32.86501, 116.59295	1085	10	69.7 ± 7.7
	Pine Creek		32.83640, 116.54304	1106	10	82.5 ± 8.5
	Kitchen Creek Canyon		32.81052, 116.44904	1528	10	67.8 ± 6.2
	Noble Canyon		32.85044, 116.52192	1134	11	78.1 ± 8.7
	Corral Canyon		32.72927, 116.54258	955	10	104.3 ± 13.3
	Guatay		32.84879, 116.55009	1215	10	62.7 ± 6.9
	Long Valley		32.81453, 116.53244	1189	10	63.4 ± 6.6
	Cottonwood		32.70152, 116.48983	953	10	72.2 ± 8.1
	Warner Springs ^b^		33.31826, 116.69494	900	10	97.4 ± 8.7
	Wooded Hill Road	*Q. kelloggii* (*Lobatae*)	32.86470, 116.45683	1692	10	89.5 ± 7.1
	Wooded Hill CG		32.84978, 116.43376	1822	10	84.1 ± 11.2
	Kitchen Creek Road		32.84705, 116.44854	1788	10	81.6 ± 9.0
	Horse Heaven		32.89007, 116.44107	1728	10	66.9 ± 8.4
	Sunrise Highway		32.84897, 116.48450	1534	10	45.7 ± 3.9
	Camp Ole		32.88230, 116.43046	1755	10	82.5 ± 6.8
	Desert View		32.87011, 116.41417	1800	10	46.9 ± 4.2
	Penny Pines		32.90527, 116.45949	1660	10	65.9 ± 9.1
AZ	Miller Canyon ^c^	*Q. hypoleucoides* (*Lobatae*)/*Q. arizonica* (*Quercus*)	31.41546, 110.27553	1744	20	32.6 ± 3.3
	Carr Canyon ^c^		31.44143, 110.28586	1658	20	22.6 ± 1.0
	Cochise Stronghold	*Q. emoryi*/*Q. hypoleucoides* (both *Lobatae*)/*Q. arizonica* (*Quercus*)	31.92567, 109.96637	1483	15	±1.7
	Chiricahua Nat’l Mon ^c^	*Q. emoryi* (*Lobatae*)	32.00880, 109.37302	1585	10	50.9 ± 3.2
	Pinery Canyon ^c^		31.96989, 109.32278	1727	10	27.5 ± 2.5
	Gardner Canyon		31.72206, 110.71739	1496	10	47.4 ± 4.4
	Madera Canyon	*Q. hypoleucoides* (*Lobatae*)	30.72500, 110.87944	1524	10	18.4 ± 1.3

^a^ Mean ± SE, measured at 1.4 m from the ground; ^b^ Site outside current zone of *A. auroguttatus* infestation in southern California; ^c^ Sites affected by the June 2011 wildfire.

AZ study sites were sampled partially during March 2011 and sampling was completed during February 2012. Wildfires affected the Chiricahua National Monument, Pinery, Miller and Carr Canyons in June 2011 [22]. Borer emergence holes were counted on three dead *Q. hypoleucoides* before the fire and seven after the fire at Miller Canyon as well as four *Q. emoryi* before the fire and six after the fire at Pinery Canyon. Density of emergence holes did not differ significantly between dead trees sampled before *vs.* after the fire at either site (pooled variance *t*-tests, *A. auroguttatus*: *t* = 1.23 and 0.26, df = 2.01 and 4.94, *p* = 0.34 and 0.80 for Miller and Pinery Canyons, respectively; and other borers: *t* = 1.15 and 0.30, df = 2.02 and 4.62, *p* = 0.37 and 0.77 for Miller and Pinery Canyons, respectively).

Climate in the Coronado National Forest in southeastern Arizona is semi-arid. Total annual precipitation varies throughout these mountain ranges from 34–58 cm, and mean August and January maximum temperatures are 32 °C and 16 °C, respectively [20]. Soils are rocky and classified mostly as Aridisols, although some are Mollisols or Entisols [23]. 

### 2.3. Evaluation of Borer Density

Cumulative borer emergence was estimated on the lower boles (≤1.52 m) of the first 10, or as many as we could find if fewer than 10, recently dead (died within the last 1–3 y, borer emergence holes were still clearly defined, and at least half of the bark on the lower bole remained) oaks encountered at all CA and AZ sites. A total of 91 *Q. kelloggii* (nine sites); 99 *Q. agrifolia* (10 sites); 10 *Q. chrysolepis* (one site); 7 *Q. engelmannii* (one site); 40 *Q. emoryi* (four sites); 33 *Q. hypoleucoides* (four sites); and 23 *Q. arizonica* (three sites) were sampled for the entire study. We did not sample living trees because our goal was to obtain a final measure of borer density from trees that were not likely to produce additional generations of borers. While some borer species may feed on dead and decaying wood, it was assumed that including only standing, recently-dead trees would limit the sample to those species that were primary or secondary mortality agents feeding on dying vascular tissue in weakened or stressed hosts. These species were most likely to interact with *A. auroguttatus* (as opposed to species that feed on dead or decaying wood).

All emergence holes were counted within a 0.09 m^2^ (1 ft^2^) sampling window (cardboard frame) at three arbitrary locations on the lower bole (≤1.52 m) of each oak tree, where *A. auroguttatus* emergence holes are dispersed randomly and occur most frequently [19]. Sampling efforts were focused on the lower bole because the goal was to estimate densities of *A. auroguttatus* where it was most likely to be present in relation to other borers. 

Emergence (and by assumption, development) by *A. auroguttatus* or other borers was determined according to emergence hole size and shape [14]. *Agrilus auroguttatus* emergence holes were D-shaped and about 4 mm wide [14,24]. The size and shape of these holes are uniquely diagnostic for this species among the community of subcortical insects on California oaks. Emergence holes from flatheaded borers (Buprestidae) other than *A. auroguttatus* (*Chrysobothris* spp.) were oblong or crescent-shaped and 5–13 mm wide; roundheaded borer (Cerambycidae) (*Rosalia funebris* (Motschulsky), *Xylotrechus nauticus* (Mannerheim) or *Neoclytus conjunctus* (LeConte)) emergence holes were round or oblong and 6–10 mm wide; false powderpost beetles (Bostrichidae) (*Scobicia declivis* (LeConte)) entrance and emergence holes were perfectly round and 4 mm in diameter (the same size as *A. auroguttatus* only perfectly round); bark and ambrosia beetles (Curculionidae: Scolytinae) (*Pseudopityophthorus* spp. or *Monarthrum* spp.) entrance and emergence holes were also round, but ≤2 mm in diameter; clearwing borer (Lepidoptera: Sessiidae) (*Synanthedon resplendens* (Edwards)) emergence holes were round and 5–6 mm in diameter and found exclusively in bark cracks near deteriorated bark and phloem; and carpenterworm (Lepidoptera: Cossidae) (*Prionoxystus robiniae* Peck) emergence holes were oval often with jagged edges and 10–20 mm wide [15,16,17,18,25,26]. Emergence holes were categorized as “unknown” if they could not be identified to insect family or if bark had deteriorated at all since the insect emerged to the extent that the hole’s original shape was somewhat unclear. There was overlap in the shape and size of emergence holes for *Chrysobothris* spp. and Cerambycidae (particularly *X. nauticus*), so counts were grouped from both taxa (bup + cer) for analyses.

### 2.4.Data Analyses

All statistical analyses were conducted within the R statistical environment, version 2.15.0 [27]. We used pooled variance *t*-tests or analyses of variance (ANOVA) to test for the effect of oak species on density of *A. auroguttatus* emergence holes. We used ANOVA to test for the effect of borer group on density of emergence holes followed by Tukey’s HSD ad-hoc tests, applied separately to each oak species at CA sites where two oak species co-occurred. 

We applied Pearson’s correlations to search for a relationship between density of emergence holes of *A. auroguttatus* and all other borer groups combined (on red oak species only), performed for each oak species (all sites combined), and separately by site. We used mixed effect linear models with restricted maximum likelihood estimation to compare number of *A. auroguttatus* emergence holes against that of all other borer groups combined, tested separately by host species for red oaks. Borer type was a fixed effect and site was a random effect.

**Figure 1:**
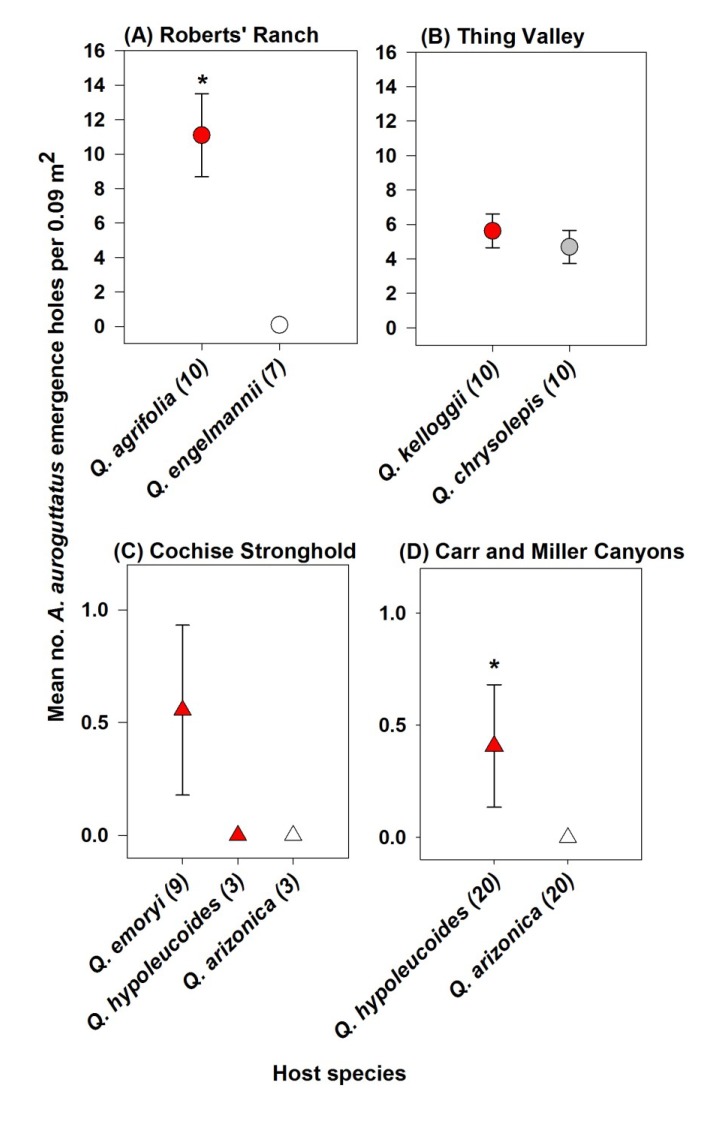
Mean densities of *A. auroguttatus* emergence holes on lower boles of dead oaks at CA sites containing host species pairs (**A**,**B**) and at AZ sites with more than one co-occurring host species (**C**,**D**). Significant differences between species are denoted by *. Sample sizes are in parentheses following species names. Oaks in Section *Lobatae* are red symbols; Section *Protobalanus* are gray symbols; and Section *Quercus* are white symbols.

**Figure 2:**
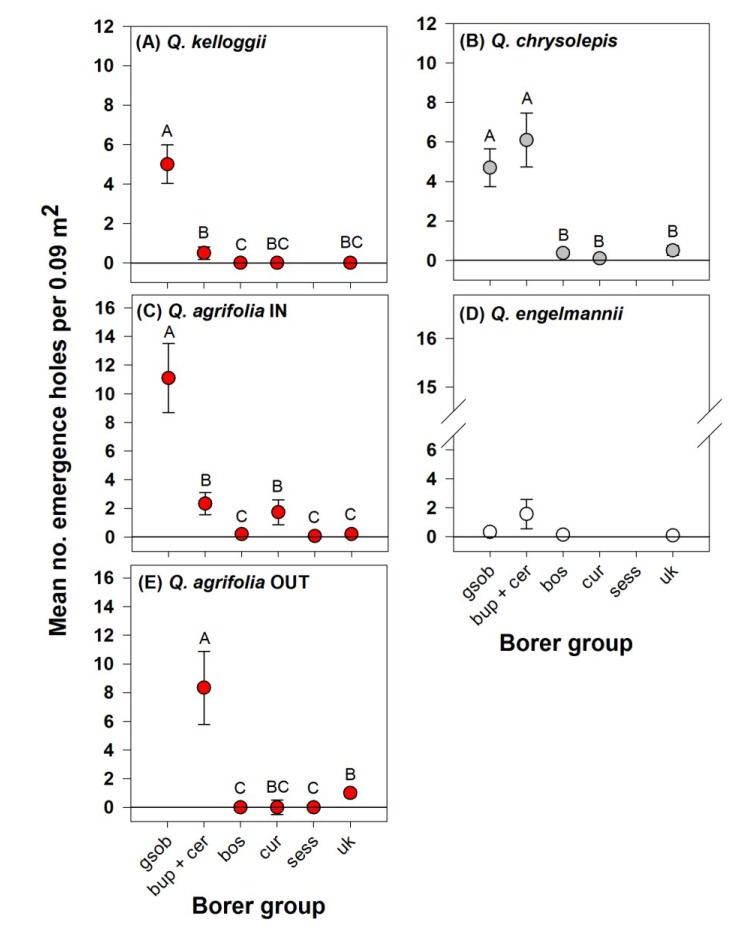
Mean densities of emergence holes on the lower boles of dead oaks by borer group, tested separately by host species at CA sites containing host species pairs (**A**–**D**) and at Warner Springs, a site outside the current zone of *A. auroguttatus* infestation in CA (**E**). Letters above bars indicate significant differences according to Tukey’s HSD; sample sizes are the same as corresponding species and sites for Figure 1; and *n* = 10 trees (**E**). Oaks in Section *Lobatae* are red circles; Section *Protobalanus* are gray circles; and Section *Quercus* are white circles. X-axis categories are as follows: gsob = *A. auroguttatus*; bup + cer = other Buprestidae combined with Cerambycidae; bos = Bostrichidae; cur = Curculionidae (Scolytinae); sess = Sessiidae; and uk = unknown.

For all linear models, model checks included the Shapiro-Wilk normality test [28], standardized residual plots, and normal probability plots. Data were transformed to meet model assumptions (adding 0.5 to original values and then applying the Box-Cox method [29] to determine appropriate transformations, used for all response data except for *t*-tests in Figure 1A,B). If data could not be transformed to meet parametric assumptions (for effect of borer group on emergence holes for *Q. kelloggii* at Thing Valley and *Q. agrifolia* at Warner Springs, Figure 2A,E, respectively), we applied ANOVA to ranks. We present results from ANOVA on ranks (Figure 2A,E) as means with standard errors for visual comparisons with corresponding parametric models of different oak species. Statistical significance was set at *p* < 0.05, all means in graphs are untransformed values, and errors are one standard error from the mean. 

## 3. Results

Density of *A. auroguttatus* emergence holes differed significantly between some, but not other, oak species (Figure 1). At Roberts’ Ranch in CA, *A. auroguttatus* density was greater on the red oak, *Q. agrifolia*, than on the co-occurring white oak, *Q. engelmannii* (*t* = 4.46; df = 9.15; *p* = 0.002; Figure 1A). *Agrilus auroguttatus* emergence holes were only found on two (mean of 1.0 ± 0.6 and 1.3 ± 0.3 per 0.09 m^2^ of bark on the two trees) of the seven *Q. engelmannii* sampled. At Thing Valley, *Agrilus auroguttatus* density did not differ significantly between *Q. kelloggii* and *Q. chrysolepis* (*t* = 0.68; df = 18.00; *p* = 0.504; Figure 1B). *Agrilus auroguttatus* density also did not differ significantly between *Q. emoryi*, *Q. hypoleucoides*, or *Q. arizonica* at Cochise Stronghold in AZ (*F* = 0.77; df = 2, 12; *p* = 0.485; Figure 1C). No *A. auroguttatus* emergence holes were found on *Q. hypoleucoides* or *Q. arizonica* at Cochise Stronghold. At Carr and Miller Canyons in AZ, *A. auroguttatus* density did not differ significantly between sites (*F* = 0.25; df = 1, 36; *p* = 0.624), but differed significantly by oak species (*F* = 21.66; df = 1, 36; *p* < 0.001; Figure 1D). *A. auroguttatus* density on *Q. hypoluecoides* was very low at these two sites, but was significantly greater than on *Q. arizonica* where no holes were found. 

At sites in CA where pairs of oak species co-occurred, *A. auroguttatus* density was significantly greater than the density of other borer groups on the lower boles of *Q. kelloggii* (*F* = 27.47; df = 4, 45; *p* < 0.001; Figure 2A) and *Q. agrifolia* within the current zone of infestation (*F* = 26.84; df = 5, 54; *p* < 0.001; Figure 2C). Bup + cer density was also relatively high on the lower bole of CA oaks. Bup + cer and *A. auroguttatus* densities were greater than density of other borer groups on *Q. chrysolepis* (*F* = 30.51; df = 4, 45; *p* < 0.001; Figure 2B). Bup + cer density was greater than densities of all other borers on *Q. agrifolia* outside the current zone of *A. auroguttatus* infestation (*F* = 36.65; df = 4, 45; *p* < 0.001; Figure 2E). Emergence hole density was very low (mean of 1.0 per 0.09 m^2^ of bark, among all borer groups), and did not vary significantly by borer group on *Q. engelmannii* (*F* = 1.76; df = 3, 24; *p* = 0.182; Figure 2D).

**Figure 3:**
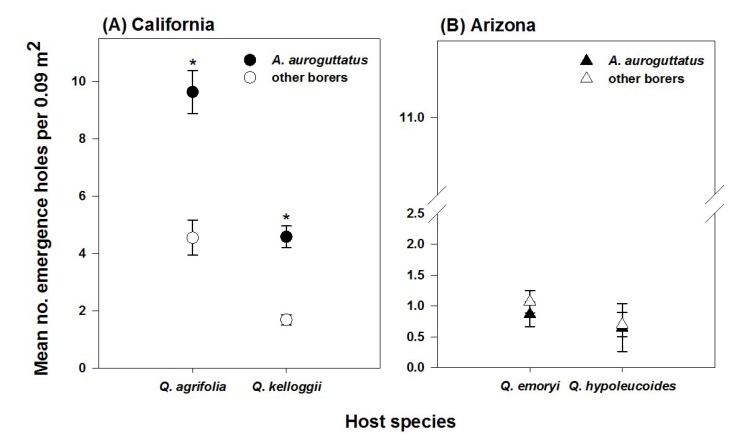
Mean densities of emergence holes from *A. auroguttatus* compared to that of other borer species combined, tested separately for each host species in CA (**A**) and AZ (**B**). * indicates significantly greater value according to restricted maximum likelihood estimation for the fixed effect borer group in mixed models. Sample sizes are: 90 trees across nine sites (*Q. agrifolia*); 91 trees across nine sites (*Q. kelloggii*); 40 trees across four sites (*Q. emoryi*); and 33 trees across three sites (*Q. hypoleucoides*).

On the lower bole, *A. auroguttatus* emergence was greater than that of all other borer species combined from red oak species across several sites in CA (*F* = 72.49; df = 1, 170; *p* < 0.001 and *F* = 70.46; df = 1, 172; *p* < 0.001; Figure 3A for *Q. agrifolia* and *Q. kelloggii*, respectively). *Agrilus auroguttatus* densities were not significantly different from those of all other borer species combined from red oak species in AZ (*F* = 2.85; df = 1, 82; *p* = 0.095 and *F* = 1.71; df = 1, 61; *p* = 0.196; Figure 3B for *Q. emoryi* and *Q. hypoleucoides*, respectively).

In general, emergence hole densities of *A. auroguttatus* were not significantly correlated with those of all other borer species combined among red oaks or at different sites. There were, however, three exceptions—emergence hole densities of *A. auroguttatus* and other borers were significantly positively correlated on *Q. hypoleucoides* among all sites (r_p_ = 0.521; *p* = 0.003; not shown), on *Q. kelloggii* at Horse Heaven in CA (r_p_ = 0.849; *p* = 0.002; not shown) and on *Q. hypoleucoides* at Miller Canyon in AZ (r_p_ = 0.995; *p* < 0.001; not shown).

## 4. Discussion

The scarcity of sites in southern CA and southeastern AZ oak forests with co-occurring oak species where *A. auroguttatus* was also present prevented some direct comparisons among oak species. Although few direct comparisons were made, this study documents patterns of emergence from natural populations of *A. auroguttatus* that suggest red oaks are either more preferable or more suitable hosts than white oaks (*i.e.*, *Q. agrifolia versus* *Q. engelmannii* and *Q. hypoleucoides versus* *Q. arizonica*; Figure 1A,D). A red oak, *Q. kelloggii*, may be equally preferable/suitable when compared with taxonomically intermediate *Q. chrysolepis* (Figure 1B). On native hosts at one site (Cochise Stronghold; Figure 1C), densities of *A. auroguttatus* on red oaks were so low that they were not significantly different from a white oak, *Q. arizonica*. At this site, neither *Q. hypoleucoides* nor *Q. arizonica* showed evidence of *A. auroguttatus* colonization. The results of this study are consistent with previous observations, which report more frequent evidence of *A. auroguttatus* colonization on red oaks than white oaks [3,13].

At the individual tree level, presence of *A. auroguttatus* in CA oak forests may result in lower densities of native buprestids and cerambycids on the lower boles of dying *Q. agrifolia*. At sites in CA with co-occurring oak species, *A. auroguttatus* was five times more abundant than all other borer groups on respective red oaks at each site (*Q. agrifolia* and *Q. kelloggii*; Figure 2A,C). Other buprestids and cerambycids were also relatively abundant on three of the four oak species examined, and more abundant than all other borer groups on *Q. agrifolia* in the absence of *A. auroguttatus* (Figure 2E).

This study focused only on the lower portion of tree boles. Community interactions elsewhere in the bole, including species partitioning with height, were not measured and may be important. For example, in eastern North American oaks attacked by *A. bilineatus*, bark and ambrosia beetles were more prevalent in upper portions of boles [30]. In that study, buprestid and cerambycid densities remained relatively constant with bole height or were greatest on the lower bole and scolytine densities were greatest at ~12.5 m above ground [30]. It is possible that *A. auroguttatus* may compete with native Buprestidae and Cerambycidae on the lower boles of dying *Q. agrifolia*, although data collected at different heights from trees of varying physiological conditions and from more sites outside the currently infested zone would be necessary to evaluate this hypothesis. 

Across several sites throughout its invaded range in CA, and on both red oaks, *A. auroguttatus* outnumbered all species of native borers combined, yet this was not the case across sites in AZ (Figure 3). Across these sites, *A. auroguttatus* densities were similar to those of all other borer groups combined, which suggests that *A. auroguttatus* does not outnumber all other borer species in its native range as it does in its introduced range. Thus, although it is a primary mortality agent of red oaks in CA, it is more likely a secondary, though relatively frequent, mortality agent of red oaks in AZ oak forests.

The mean and range densities of *A. auroguttatus* that emerged from susceptible naïve hosts varied somewhat from those reported in other studies of *Agrilus* species on their respective naïve hosts (Table 2). The range that we reported for *A. auroguttatus* on *Q. agrifolia* was influenced by one tree that contained 50 emergence holes per 0.09 m^2^, the next greatest density was only 29 emergence holes per 0.09 m^2^. Similar to our observations of *A. auroguttatus* on co-evolved hosts, emergence densities of other *Agrilus* species were much lower on co-evolved than naïve hosts (Table 2). Many factors undoubtedly varied among the other studies referenced in Table 2 and ours. However, in those studies, trees had died recently or were moribund, and emergence densities reported were likely a result of the cumulative number of *Agrilus* beetles produced by dying trees.

**Table 2:** Mean (range) emergence hole density of *Agrilus* spp. on co-evolved and naïve host trees reported from the literature and this study.

Agrilus spp.	Host spp.	Relationship	Density per 0.09 m^2^ of bark	Density per m^2^ of bark	Reference
*A. auroguttatus* Schaeffer, goldspotted oak borer	*Quercus agrifolia*	naïve	10(1–50)	111(11–556)	This study ^a^
	*Q. kelloggii* (both *Fagaceae*)	naïve	5(0–16)	56(0–178)	
*A. planipennis* Fairmaire, emerald ash borer	*Fraxinus pennsylvanica*, *F. americana* (Oleaceae)	naïve	8(2–15)	89(17–170)	[31] ^b^
*A. anxius* Gory, bronze birch borer	*Betula pendula* (Betulaceae)	naïve	21(N/A)	232(N/A)	[32] ^c^
*A. auroguttatus*	*Q. emoryi*	co-evolved	1(0–7)	11(0–78)	This study
	*Q. hypoleucoides*	co-evolved	1(0–12)	1(0–133)	
*A. bilineatus* (Weber), twolined chestnut borer	*Q. rubra*, *Q. velutina*	co-evolved	4(N/A)	41(N/A)	[33] ^b^
*A. biguttatus* (Fabricius), oak buprestid beetle	*Quercus* spp.	co-evolved	Max = 7	Max = 76	[34] ^d^

^a^ Trees died 1–3 y before study; ^b^ trees died 1 y before study; ^c^ trees had died recently; ^d^ heavily infested trees.

## 5. Conclusions

Sites with co-occurring oak species that also contained populations of *A. auroguttatus* were scarce, which made testing the role of host species in within-tree densities of *A. auroguttatus* difficult. Nonetheless, in a small number of white oaks surveyed in CA and AZ, few *A. auroguttatus* emergence holes were recorded, whereas at some locations, significantly higher emergence hole densities were recorded for various red oaks. As an invasive species in CA, *A. auroguttatus* may affect the community of native borers feeding in the lower boles of oaks, although it remains unclear whether its impact will be positive or negative. One possibility is that primary attack by *A. auroguttatus* could weaken trees and create more suitable hosts on the landscape for native borer populations. This does not seem to be the case, since we did not find much evidence of a positive correlation between emergence hole densities for *A. auroguttatus* and other borers among oak species or sites. Alternatively, by attacking apparently healthy trees and colonizing them for several successive generations, *A. auroguttatus* may out-compete native borers in dying trees. Both of these possibilities should be investigated further in CA and AZ oak forests. 
